# A Difficult Differential Diagnosis in New Neck Masses: Retropharyngeal Abscess or Malignancy?

**DOI:** 10.7759/cureus.61895

**Published:** 2024-06-07

**Authors:** Mary Therese Thomas, Mary Carter

**Affiliations:** 1 Internal Medicine, Grand Strand Medical Center, Myrtle Beach, USA

**Keywords:** radiology & imaging, oral maxillofacial radiology, guidelines in medicine, emergent airway, partial airway obstruction, antibiotic treatment, drug susceptibility testing and antibiotic resistance, delayed diagnosis, retropharyngeal abscesses

## Abstract

Retropharyngeal abscesses (RPAs) are rare in the adult population and rarer without an inciting event or comorbidity such as recent oral surgery, neck infection, or pharyngeal trauma. The definitive treatment is incision and drainage of the abscess. Clinical researchers have recently questioned whether invasive surgical intervention is necessary and posed the question of what role antibiotics play in management. Sequelae of RPAs are severe and include rupture of the abscess, erosion of the carotid artery, thrombophlebitis, and most seriously, airway compromise. We present a case where an atypical presentation of an RPA caused a disagreement among specialists, and the debate of whether the described case represented an abscess or malignancy caused a delay in diagnosis and treatment for the patient. Only after invasive and emergent surgical intervention was a final diagnosis able to be made. This case demonstrates the need for more research and official guidance on the management of new neck masses to hasten diagnosis and prevent devastating outcomes.

## Introduction

Retropharyngeal abscesses (RPAs) are rare in the adult population and rarer without an inciting event or comorbidity. It is hypothesized that as we age, we have a decreased risk of having an RPA due to atrophy of our retropharyngeal lymph nodes and the fact that we contract fewer respiratory infections [1]. Predisposing conditions, such as recent oral or dental surgery, head and neck infection, or pharyngeal trauma from a foreign object, are notable risk factors for the development of an RPA [2-4].

The most common presenting symptoms of an RPA are dysphagia, neck pain, and sore throat [3]. It is difficult to diagnose an RPA by physical exam alone. As can be expected, the patients may have difficulty moving their necks and opening their mouths, which makes visualization of the posterior pharynx difficult. On physical exam, noted findings may include cervical lymphadenopathy, neck stiffness or swelling, and stridor [5]. The neurologic exam is often normal [2]. Any of these symptoms, but, most importantly, pain out of proportion to the exam, should be concerning and prompt emergency evaluation [3]. The definitive treatment is incision and drainage of the abscess, but clinical researchers have recently questioned whether invasive surgical intervention is necessary [6,7]. Antibiotics alone have been proven to be effective, but without aspiration of pus, culture speciation and antibiotic sensitivity testing cannot be completed. The subsequent empiric prescription of broad antibiotics contributes to antibiotic overuse and organism resistance [6-8]. If not promptly diagnosed and treated, the sequelae of RPA include rupture of the abscess, erosion of the carotid artery, jugular thrombophlebitis, thrombosis of the cavernous sinus, and most seriously, airway compromise [3].

We present an atypical case of RPA in an immunocompetent male with no predisposing comorbidities or conditions and without inciting factors or otherwise known risk factors for an RPA. Atypical presentation and conflicting provider opinions made the diagnosis difficult. We hope that by presenting this case, we can facilitate interest in this difficult differential and demonstrate the need for improved official guidance to hasten diagnosis and prevent devastating outcomes for these patients.

## Case presentation

We present the case of a 51-year-old male who came to our emergency department after approximately two weeks of left-sided neck pain and temporal headache. The patient had a minimal past medical history, which included anxiety and depression, testicular cancer status post orchiectomy in 2005, and multiple lumbar back surgeries. Two weeks before the visit to our emergency department, he was examined and treated at another emergency department. There, he had multiple computed tomography (CT) scans, which were nonrevealing, and an occipital nerve block, which did not relieve his pain. He was discharged from the emergency room with cyclobenzaprine hydrochloride, prednisone, and oxycodone/acetaminophen which provided minimal relief. The patient’s presentation was associated with left eye blurriness, difficulty mobilizing the jaw, and dysphagia. He denied any fever, chills, dizziness, diplopia or loss of vision, angina, palpitations, shortness of breath, abdominal pain, nausea/vomiting, weight loss, hemoptysis, diarrhea, or urinary symptoms. He did not have any numbness or tingling in the upper or lower extremities, recent illness, upper respiratory infection, or dental infection. He had not traveled recently but he did endorse a history of frequent streptococcal tonsillitis without recent episodes nor a history of abscess. The patient was a previous smoker of one pack per day for an unknown number of years but quit >20 years ago. He continued to use chewing tobacco.

On examination, the patient was afebrile with a temperature of 98.8°F, pulse rate of 98, respiratory rate of 18, blood pressure of 158/93, and oxygen saturation of 98% on room air. He was in severe distress due to pain and was sitting at the edge of the bed with his head tucked between his legs, wrapped in a blanket. The patient was alert and oriented to person, place, and time. He was unwilling to participate in much of the history taking or exam due pain-related “inability to think”. The head was atraumatic and normocephalic. A sharp stabbing pain was able to be reproduced with a light touch over the left occipital region and distribution of all branches of the left trigeminal nerve. There was severe photophobia in both eyes, but pupils were equal and reactive. The temporal artery was not swollen and no meningeal signs were noted. Visual examination of the oropharynx was limited due to the patient's inability to open his mouth widely. The mucous membranes were moist. We were not able to visualize the tonsils, posterior pharynx, or uvula during the examination. The tongue was of normal caliber and projected midline. The neck was non-tender without obvious lymphadenopathy or notable masses. Examination of the heart, lungs, abdomen, and extremities were normal.

On initial laboratory evaluation, white blood cells, platelets, C-reactive protein (CRP), and erythrocyte sedimentation rate (ESR) were all elevated (Table 1).

**Table 1:** Notable lab values on admission Higher than normal range lab values are indicated with an "H".

	RESULT		RANGE
White blood cells	19.8 K/mm3	H	3.7-10.1 K/mm3
Platelets	556 K/mm3	H	156-352 K/mm3
C-reactive protein (CRP)	19.9 mg/dL	H	0-0.99 mg/dL
Erythrocyte sedimentation rate (ESR)	62 mm/hr	H	0-15 mm/hr

**Figure 1:**
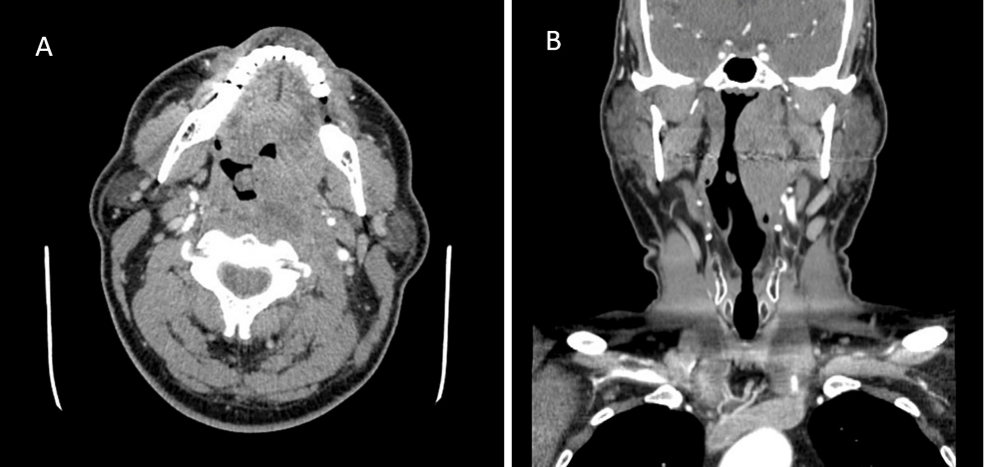
Initial CT head and neck performed on admission showing concern for airway-compromising mass, abscess versus malignancy

**Figure 2:**
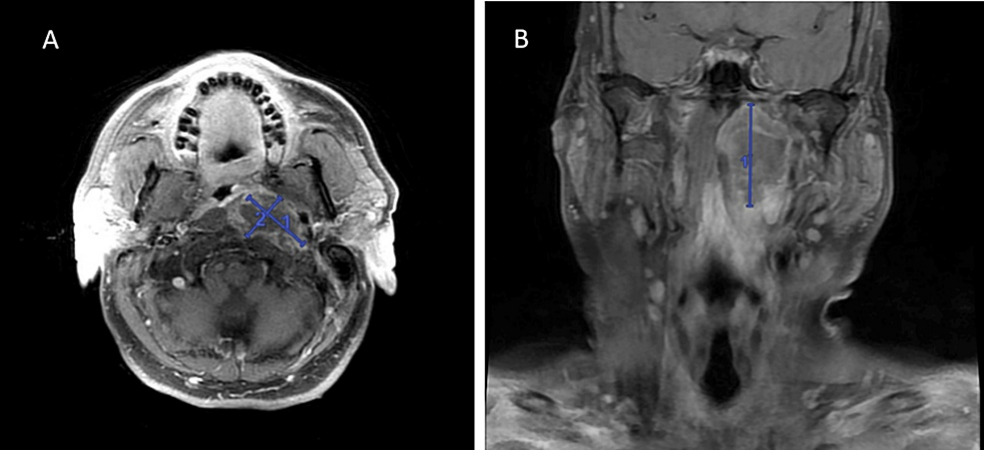
MRI head and neck trans-spatial, rim-enhancing fluid collection centered within the left parapharyngeal space, measuring 51 x 38 x 27 mm

Cerebrospinal fluid (CSF) studies, including cultures, were within normal ranges and negative for all viruses or bacteria tested. CT angiography of the head and neck was read as an “edematous left palatine tonsil resulting in mild rightward airway displacement and effacement. The lesion was of central low attenuation without peripheral enhancement, suggestive of more focal edema or early abscess formation (Figure 1). Because these results were largely inconclusive, a magnetic resonance image (MRI) of the orbits/face/neck was ordered. The MRI was interpreted as a trans-spatial rim-enhancing fluid collection centered within the left parapharyngeal space, measuring 51 x 38 x 27 mm (Figure 2). Although closely associated with the palatine tonsils, the mass did not appear to be centered or originating from the palatine tonsil or pharyngeal mucosa. There was an associated mass effect on the displaced adjacent structures, including compression and at least partial obstruction of the left internal jugular vein, without clear invasion. It was noted on this MRI interpretation that an infectious etiology could not be ruled out, but there was a high suspicion of primary malignancy, most likely nasopharyngeal carcinoma, and a CT with contrast of the same anatomical area was recommended for definitive imaging and nodal staging. This CT with contrast was ordered and interpreted to, again, be an infiltrative, trans-spatial mass favored representing nasopharyngeal carcinoma “extending through the masticator, parapharyngeal, retropharyngeal, and perivertebral spaces and with inferior extension through the palatine fossa, soft palate, and vallecula” with “prominent bilateral cervical lymph nodes with perinodal fat stranding” suspicious for nodal involvement of malignancy.

We started the patient on IV ampicillin-sulbactam 3 g every six hours one hospital day one. Multiple efforts were made in attempts to control his pain, which included administration of gabapentin, nasal oxygen therapy, ketorolac, and codeine derivatives, all of which were ineffective. The ear, nose, and throat (ENT) surgeons were consulted and ultimately took the patient to the operating room on hospital day three for further exploration of the retropharyngeal space and visualization of the lesion due to concern for impending airway compromise in the setting of mass effect. A copious amount of bright yellow-colored purulence was able to be expressed from the posterior left palatine tonsillar fossa. This purulence was sent for cytology and microbiology culture. During this operation, an occipital nerve block was completed using 5 mL of 0.25% bupivacaine and 4 mg of dexamethasone.

**Figure 3:**
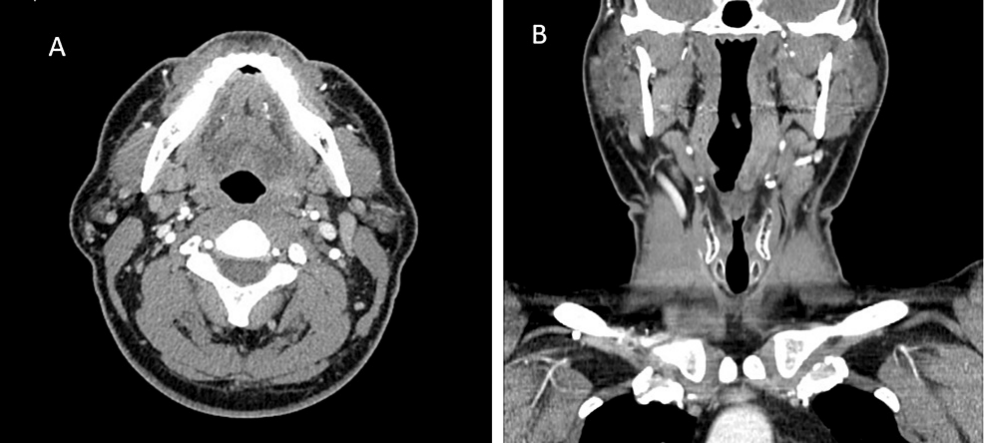
One month post incision and drainage, CT head and neck shows resolution of the previously observed retropharyngeal abscess with minimal residual changes of the oropharynx

Postoperatively, the patient recovered well. The pain was better controlled, and the oral pain regimen was able to be de-escalated to acetaminophen and gabapentin, as needed. He passed a swallow evaluation with speech therapy and clinically improved. Cytology returned negative for malignant or dysplastic cells. Gram stain and culture grew methicillin-resistant Staphylococcus aureus. He was continued on ampicillin-sulbactam 3 g intravenously (IV) every six hours and daptomycin 900 mg IV daily for a total of three weeks after source control per the recommendation of the Infectious Disease specialists. At a follow-up appointment with the ENT surgeons one month following incision and drainage and discontinuation of antibiotic therapy, repeat neck CT with contrast showed resolution of previously observed retropharyngeal abscess with minimal residual changes of the oropharynx (Figure 3).

## Discussion

Limited official guidelines exist on the management of RPAs. The most recent guidelines were published in 2017 by Pynnonen et al. for the American Academy of Otolaryngology. These authors attempted to hasten the diagnosis of head and neck cancer, noting that the diagnosis of a new neck mass takes an average of three to six months [9]. While the Pynnonen et al. guidelines focused mostly on neoplastic masses, many of the initial diagnostic steps for a new neck mass are the same for infectious masses.

Our patient is a prime example of this delay in the diagnosis of new neck masses. He did not present as septic or with any concern for an infectious process. Only after visiting two different hospitals and undergoing numerous imaging studies and various treatment approaches, all of which provided no definitive resolution, did he finally meet critical criteria for diagnostic exploration of this mass due to impending airway compromise. It is unclear what was discovered at the outside hospital, whether the clinical picture worsened, or perhaps the mass size rapidly increased over the course of the two weeks, causing him to continue seeking medical care. Either way, left-sided neck pain has a wide differential, which may explain why limited medical treatment was given. Prior to imaging at the time of presentation to our hospital, the differential diagnosis included trigeminal neuralgia, giant cell arteritis, migraine, cerebrovascular accident, and meningitis. Even with imaging, there was no obvious distinguishable area of fluctuance or other clinical characteristics that pointed toward an infectious process. With multiple concerning diagnoses, a detailed history was obtained from this patient in an attempt to help support one diagnosis over the other. Risk factors for nasopharyngeal carcinoma include Southern Chinese ethnicity, lifetime intake of preserved foods, tobacco use, and occupational exposure to formaldehyde and wood dust [10]. Out of these, only tobacco use applied to our patient. Once again, clinical history could not support a diagnosis of nasopharyngeal carcinoma over infectious abscess. Only after direct visualization in the operating room was the abscess undeniably diagnosed.

Some specialists agree that in the setting of impending airway compromise, aggressive management with elective intubation and incision and drainage is a preferred method over traditional antibiotic management, but extremely limited data exists on this topic [8]. Pynnonen et al. recommend that, unless there are signs and symptoms of bacterial infection, antibiotic therapy should not be routinely prescribed [9]. They too conclude that guidance on the role of antibiotics in new neck masses is an area where literature is lacking [9].

Perhaps one of the newest markers being studied to help differentiate the infectious neck processes is pro-calcitonin (PCT). PCT is the precursor to calcitonin, the hormone that manages calcium regulation in the body and has previously been used to simply identify the presence of bacterial infections. Yankov and Bocheva note that along with the traditionally studied values of inflammation such as elevated white blood cell counts and C-reactive protein (CRP), PCT was elevated in their retrospective study of men with active purulent odontogenic infections [11]. The physiologic process behind this is that as bacteria begin to form the infection, many cells in the body, beyond just the parathyroid, begin to synthesize PCT at a rapid rate [11]. In comparison to other values, PCT appears to become elevated much earlier in the disease course (peak at two hours versus five for CRP) and decreases quicker after the resolution of the infection [11]. Although studied strictly in patients with odontogenic infections as the nidus of infection, this relatively cheap and quick test may be the answer to a noninvasive diagnosis in patients like ours.

## Conclusions

This case broadens the differential of severe left-sided facial pain in those with pain out of proportion to the exam. Our initial examination of this patient led us to explore more inflammatory causes of his pain, versus infectious, given a largely benign exam. Only after imaging showed concern for airway compromise was a final diagnosis of an RPA made. In this case, particularly, the patient had no initial difficulty with breathing, no risk factors for the development of an RPA, and no prior inciting events, which made the diagnosis of an RPA likely. Finally, the difference in opinions of specialist providers delayed treatment. Though the patient ultimately underwent surgical incision and drainage, the question of whether he needed an invasive operation for treatment stands and whether conservative medical management would have sufficed. We can assume that all of these “pro-surgery benefits” may not have been the case in others with an RPA. While the outcome for our patient was favorable, he is an excellent example of the necessity for the prompt diagnosis of and guideline-directed management of retropharyngeal abscesses in adults.
